# Metalloproteinase dependent reduction of cell surface cluster determinants upon the induction of apoptosis

**DOI:** 10.3892/ijo.2014.2344

**Published:** 2014-03-13

**Authors:** ALBERT MAGRO, ALICE MAGRO, SIRISH SHRESTHA, KATHY BRUNDAGE, GARY RANKIN

**Affiliations:** 1 Department of Biology, Fairmont State University, Fairmont;; 2 Departments of Statistics, West Virginia University, Morgantown;; 3 Microbiology, Immunology and Cell Biology, West Virginia University, Morgantown;; 4 Department of Pharmacology, Physiology and Toxicology, Joan C. Edwards School of Medicine, Marshall University, Huntington, WV, USA

**Keywords:** apoptosis, cluster determinants, metalloproteinases, glioblastoma, growth factor receptors, integrins

## Abstract

LN18 glioblastoma cells were used as a model to examine changes in surface cluster determinants (CDs) as the cells undergo apoptosis. LN18 cells proceeding through apoptosis manifested a decrease in cell adhesion molecules, growth factor receptors and other surface proteins. Apoptosis was induced by MK886, a known FLAP and PPAR-α inhibitor, or staurosporine, a known inhibitor of protein kinases including protein kinase C (PKC). The detection and decrease of surface CDs were observed by flow cytometry using CD-specific primary antibodies followed by secondary antibodies conjugated to phycoerythrin. It was determined that there was an apoptotic induced decrease of α and β integrin determinants and the growth factor receptors EGFR and IGF1R. The MHC-1 cell surface marker HLA-ABC was also reduced in the apoptotic cells. The level of EGFR, IGF1R and detected α and β integrin determinants dropped dramatically. The degradation takes place in mid to late apoptosis. It was determined by real-time RT-PCR that the decrease in integrins, EGFR, IGF1R and MHC-1 determinants were not due to a reduction in transcription. Inhibitors of metalloproteinases blocked the apoptotic decrease in cell surface determinants indicating that metalloproteinases mediated the reduction in these CDs in a manner that can reduce growth and survival signals while stimulating the NK surveillance system. Overall, the data indicate that the final stages of the pharmacological induction of apoptosis, while proceeding to a full commitment to non-necrotic cell death, involves the degradation of integrin, insulin and epidermal growth factor receptors caused by a programmed dysregulation of the cell’s metalloproteinases.

## Introduction

Programmed cell death was first recognized as a process of tissue restructuring (1) which led to the understanding that it is a gene-guided progression that occurs naturally particularly in the embryonic development of tissues, organs and limbs during the growth and development of an organism (2–5). This type of cell death is known as apoptosis and has been recognized as an active regulatory mechanism complementary to, but functionally opposite of, proliferation. Death programs are also initiated in the somatic cells of mature organisms for the purposes of tissue turnover and the eliminating of abnormal cells. Encoded progressions are intrinsic to various programmed cell death processes which include autophagy (6), apoptosis (7), anoikis (8) and possibly aspects of necrosis (9,10). Cell death progressions can be initiated by a variety of stimuli including free radicals, hypoxia, hyperthermia, radiation, viral infections, toxins and cytotoxic pharmacologic agents. These direct stimuli initiate programmed cell death by what is referred to as intrinsic signaling pathways. Intrinsic pathways are resisted by growth promoting cytokines and the stimulation of growth factor and integrin receptors that normally promote cell survival and simultaneously suppress death programs. Conversely, intrinsic cell death progressions can be initiated or assisted by the absence of required survival signals. Thus, cells obtain survival signals via extracellular stimuli provided by matrix proteins and growth factors within the cell’s microenvironment. Survival signals are initiated via cell surface receptors capable of implementing transduction mechanisms which result in the recruitment and activation of intracellular effector proteins. The transduction mechanisms of the integrin family of receptors (11) along with insulin type-1 (IGF1R) and epidermal growth factor (EGFR) receptors have been particularly well studied (12,13) and there is an emerging picture of interrelationships that exist between their intracellular signaling systems (14,15).

The term apoptosis was coined in 1972 by Kerr *et al* (16) and is the most commonly used term to describe a form of programmed cell death that is distinct from autophagy and necrosis. Anoikis is a particular form of apoptosis induced by the disruption of integrin mediated cell-matrix interactions (17). Integrins constitute an important cell surface system that provides cells with anchorage and growth properties (18,19). The disruption of anchorage-dependent cell growth mechanisms was quickly realized to be an initiator of anoikic pathways (20,21). Anoikis and apoptosis together are important aspects of controlling cancer progression. It is well known that non-necrotic radiological and pharmacological treatments of tumors induce cell death primarily by apoptosis (22). There is considerable interest in the resistance of cancer cells to anoikis (23), along with resistance to drug/radiation induced apoptosis, particularly in the context of metastases, invasiveness and therapeutic regimens in a variety of cancer cell types (24–26). Although there may be a continuum of biochemical and cytomorphological changes when comparing apoptosis to necrosis (27), cells undergoing apoptosis manifest some morphological changes that are distinguishable from necrosis (28). Morphological changes that are characteristic of apoptosis include cell shrinkage, chromatin condensation, blebbing at the cell surface with an intact plasma membrane, and nuclear fragmentation that is contained within the cell or within the apoptotic blebs of the cell. As apoptosis progresses the population of apoptotic cells can lose cell-to-cell adhesions and will separate from neighboring cells and the extracellular matrix. This raises the question of whether there is a reduction in the transcription/translation of integrin receptors, as cells undergo apoptosis. Alternatively, the loss of integrin determinants may involve an enzymatic degradation by cell sheddases that are activated by the apoptotic process. Using the LN18 glioblastoma cell line as a model, we investigated whether integrins, growth factor receptors and MHC-1 determinants are modified as cells proceed throughout the process of apoptosis.

## Materials and methods

### Cell type and culture conditions

The LN18 cell line (ATCC, CRL-2610) was established in 1976 from a patient with a right temporal lobe glioma. The cells are poorly differentiated, adherent and grow well in culture (29). LN18 cells were maintained in Dulbecco’s modified Eagle’s medium, free of phenol red and supplemented with the dipeptide L-alanyl-L-glutamine (2 mM), non-essential amino acids, pyruvate (100 *μ*g/ml), penicillin (100 U/ml), streptomycin (100 *μ*g/ml), amphotericin B (0.25 *μ*g/ml), HEPES (25 mM), and fetal bovine serum (10%), at 37°C in an atmosphere of 5% CO_2_. Cells were subcultured by trypsinization (0.25% trypsin, EDTA). All reagents were purchased from Sigma/Aldridge or Invitrogen.

### Apoptotic inducing agents

MK886 (50 *μ*M) and stauro sporine (1 *μ*M) were used as apoptotic inducing agents. MK886 induces apoptosis in a variety of cancer cells (30–33) and inhibits the action of five lipoxygenase activating protein (FLAP) and blocks the formation of leukotrienes generated by the ALOX-5 pathway (34–36). Staurosporine inhibits a variety of kinases including protein kinase C (37) and is a proven apoptotic inducing agent (38,39).

### Monolayers of LN18 cells assayed for apoptosis by Annexin V binding, changes in mitochondrial potential, TUNEL and release of soluble DNA-histone complexes

Apoptosis was demonstrated by established tests including: morphological changes, release of histone-associated-DNA-fragments from the nucleus into the cytoplasm, Annexin V binding to membrane exposed phosphatidylserine, changes in mitochondrial membrane potential and the TUNEL assay. For examination by fluorescent microscopy, cells were plated onto 8 chambered glass slides (Lab Tech II) at 2×10^4^ cells/chamber. Following adherence and treatment with apoptotic inducing agents, the treated and DMSO vehicle control cells were fixed with 0.1% paraformaldehyde-PBS for 15 min at room temperature, washed and photographed digitally.

To assay for surface phosphatidylserine exposure cells were stained with Annexin V-488 and PI per manufacturer’s instructions (Roche, Annexin V-FLOUS kit). All flow cytometry samples were assayed on the same day using a two laser, 4 color FACSCalbur (BD Biosciences, San Jose, CA) with a minimum of 10,000 events per sample. Changes in the mitochondrial function were detected by changes in fluorescent intensity of the mitochondrial membrane binding dye Mito Tracker Deep Red 633 (Molecular Probes). Cells were stained live at 37°C in 300 nM of Mito Tracker for 20 min. Cells were harvested and then fixed in 1% paraformaldehyde and subsequently analyzed by flow cytometry. The TUNEL assay was performed on treated and DMSO vehicle control cells according to the manufacturer’s instructions (Invitrogen). Cells were washed and fixed with 1% paraformaldehyde then permeabilized with 70% ethanol. The fragmentation of nuclear DNA was detected by terminal deoxynucleotidyl transferase (TdT)-mediated dUTP nick end-labeling (TUNEL). The incorporated BrdU was immunocytochemically detected by anti-Br-dU antibody conjugated with Alexa-488 dye. Once the cells were labeled with anti-Br-dU antibody conjugated with Alexa-488 dye, the samples were assayed by flow cytometry.

### Release of soluble DNA-histone complexes

Release of soluble DNA-histone complexes into the cytosol were detected in 96-well plates by an ELISA technique. LN18 cells were plated in 96-well microtiter plates (1×10^4^ cells/well). The cells were allowed to adhere overnight and then treated with the indicated concentrations of MK886 or staurosporine for the time periods indicated. Cells were carefully rinsed and permeabilized by adding lysis buffer (Roche). After centrifugation, supernatants of the permeabilized cells were transferred to streptavidin-coated 96-well microtiter plates and tested for DNA-histone complexes by ELISA using anti-histone-biotin antibody followed by peroxidase conjugated anti-DNA using 2,2′-azino-bis (3-ethylbenzthiazoline-6-sulfonic acid) as substrate (Roche Cell Death Detection ELISA kit). The development of product was measured in a Dynatech Microplate Reader at 405 nm with a reference wavelength at 490 nm.

### Cellular surface protein levels determined by flow cytometry

Flow cytometry fluorescence intensity changes enabled the determination of changes in levels of integrin receptors, growth factor receptors and other cell surface cluster determinants in non-apoptotic LN18 cells as compared to apoptotic LN18 cells. Monolayers (90% confluent) of normal and MK886 or staurosporine treated monolayers of LN18 cells were lifted from the 75 cm^2^ flasks by treatment with a non-enzymatic cell dissociation buffer (Gibco). The cells were then washed with ice cold PBS that was 0.5% in BSA, 1 *μ*g/ml of purified human IgG and then centrifuged at 400 × g for 10 min. Washed cells (100 *μ*l) (1×10^5^ cells) were then reacted with purified rabbit primary antibodies (5 ng/*μ*l) or mouse primary antibodies (5 ng/*μ*l) directed against specific determinants for 30 min on ice. The following antibodies were used as negative isotype controls: rabbit anti-KLH (Sigma) and Mouse anti-KLH (Biolegend). The following mouse anti-human primary antibodies were used: anti-EGFR (Abcam), anti-IGF1R (Affymetrix), anti-HLA-ABC (Biolegend), anti-integrin α2 (BD Biosciences) and anti-integrin β3 (BD Biosciences). Polyclonal rabbit anti-human MMP3 was purchased from Sigma. The cells were then washed and centrifuged at 250 × g for 10 min with ice cold PBS that was 0.5% in BSA. The cells were subsequently incubated in a volume of 100 *μ*l for 20 min with phycoerythrin conjugated goat anti-rabbit IgG (Jackson ImmunoResearch) or phycoerythrin conjugated anti-mouse IgG (Jackson ImmunoResearch) at a reaction concentration of 1.5 ng/*μ*l. The cells were then washed 2x with PBS (0.1% normal goat serum), fixed in 1% paraformaldehyde, washed and assayed by flow cytometry.

### Real-time RT-PCR

Total cellular RNA was isolated using TRIzol reagent, following manufacturer’s instructions (Life Technologies). Reverse transcriptase generated c-DNA(s) were obtained using random hexamers with the high capacity archive kit (Applied Biosystems) from RNA concentrations of 6.25 ng/*μ*l. The c-DNA(s) were allowed to form for 2 h at 37°C. Negative controls were generated by omitting the reverse transcriptase in the cDNA-generating step. For the PCR step, the primers and Taq-Man fluorescent probes were purchased from Applied Biosystems. The primers were designed to span an intron to avoid amplification of any contaminating DNA. Real-time RT-PCR was performed using the Applied Biosystems Gene Amp 5700 system with the Taq-Man Universal PCR Master Mix. Relative mRNA levels were measured using the cycles to threshold (Ct) method, defined as the cycle number that first gives detection of the PCR amplicons above a fixed threshold baseline set within the log phase of the plot of fluorescence versus cycle number. There were 4 replicates for each sample. The amplicons were generated over 40 cycles where each cycle consisted of a 15 sec dissociation step at 92°C and a polymerization step at 60°C for 1 min. The changes in Ct values (ΔCt) for the housekeeping gene β-actin were obtained by subtracting the Ct value of the vehicle (DMSO) control cells from the Ct value for the MK886 treated cells. The ΔCt values for the genes of interest were similarly obtained. A normed (ΔΔCt) was calculated for each sample by subtracting the ΔCt value of the housekeeping gene β-actin (ACTB) from the ΔCt value for the gene of interest. Samples were assayed by ANOVA followed by a Tukey test with a p-value <0.05 accepted as a significant difference.

## Results

### MK886 and staurosporine induced apoptosis manifest typical morphological changes

**Figure 1.**
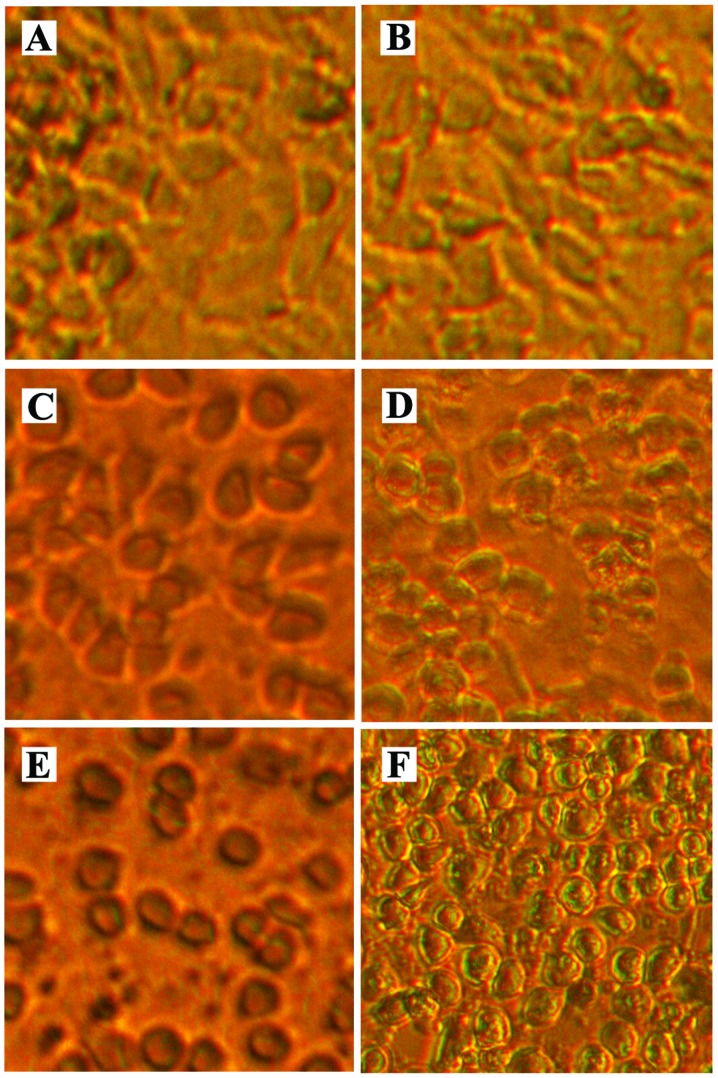
Rounding and shrinking of LN18 cells as they progress through apoptosis. Monolayers of DMSO vehicle control LN18 cells (A and B) were treated with 1 *μ*M of staurosporine for 3 h (C) and 6 h (E). D and F were treated with 50 *μ*M of MK886 for 3 and 6 h, respectively. Comparing the panels of micrographs (×200) illustrates the rounding of LN18 cells as they progress through apoptosis.

Fig. 1A and B are micrographs of a confluent monolayer of DMSO vehicle control LN18 glioblastoma cells. The micrographs of the DMSO vehicle control monolayers illustrate that there is contact between neighboring cells. Micrographs showing treatment with 1 *μ*M of staurosporine (Fig. 1C and E) or 50 *μ*M of MK886 (Fig. 1D and F), each for 3 and 6 h, illustrate that monolayers of LN18 cells manifest the morphologic change of rounding and shrinking as they proceed through apoptosis. It can be seen that by 3 h of treatment with staurosporine the cells round up and are separate from each other. By 6 h, the MK886 treated cells are also clearly separate from each other resulting in a decrease in cell/cell integrin signaling and consequently a decrease in survival signaling. As the apoptotic process proceeds the cells shrink further, form cell surface blebs and separate from the extracellular matrix which further decrease integrin mediated survival signaling (not shown).

### Translocation of phosphatidylserine that is characteristic of apoptosis

**Figure 2.**
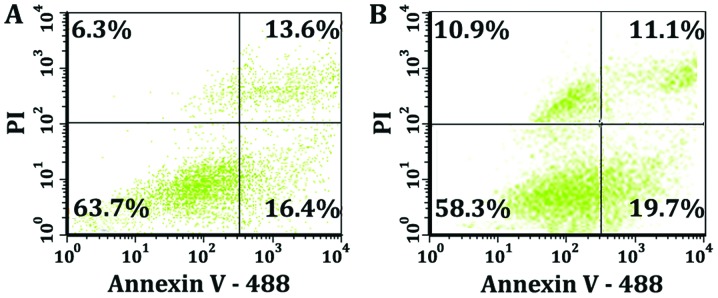
Dot plots for LN18 cells treated with staurosporine or MK886. LN18 cells in a monolayer were treated with 50 *μ*M of MK886 (A) and 1 *μ*M of staurosporine (B) for 8 h. Following incubation with inducing agent the cells were harvested, labeled with Annexin V-488 and propidium iodide, and analyzed by flow cytometry. Numbers denoted in quadrants of each plot represent the percentage of cells in each quadrant. Viable cells that are not positive for Annexin V-488 or propidium iodide are neither apoptotic nor necrotic and are represented in the lower left quadrant; necrotic cells devoid of apoptosis that stained positive for propidium iodide, but not for Annexin V-488 are represented in the upper left quadrant; apoptotic cells devoid of necrosis and stained for Annexin V-488, but not propidium iodide are in the lower right quadrant; and, late apoptotic/necrotic cells that are stained for both Annexin V-488 and propidium iodide are represented in the upper right quadrant.

Detecting exposure of phosphatidylserine on the outer leaflet of the plasma membrane is one of the classic tests for apoptosis. Phosphatidylserine exposure in the absence of plasma membrane rupture indicates a state of apoptosis that is devoid of necrosis. The most common test to separate populations and determine the fraction of cells that are normal, pure apoptotic, apoptotic/necrotic or necrotic is to generate dot plots and analyze multiple colors of fluorescence with respect to each other. The dot plots of Fig. 2 show the intensity of fluorescence of cells that express Annexin V-488 binding (abscissa) in comparison to the intensity of fluorescence of cells that uptake the nuclear binding dye propidium iodide (ordinate). The quadrants of Fig. 2A demonstrate that at 8 h there is a mixed population of cells when they are treated with 50 *μ*M of MK886 for 8 h. The lower right quadrant of Fig. 2A shows that 16.4% of the total population express only Annexin V-488 binding illustrating the fraction of cells that are pure apoptotic and devoid of any plasma membrane disruption. The lower left quadrant of Fig. 2A shows that population (63.7%) that has not progressed to phosphatidylserine exposure or propidium iodide (PI) binding. The pharmacological induction of apoptosis *in vitro* typically progresses into a population that is apoptotic/ necrotic and finally necrotic. This is demonstrated by the upper right quadrant of Fig. 2A which shows that 13.6% of the cells of the population express both PI and Annexin V-488 while the upper left quadrant 6.3% of the cells of the population express PI only. The data of Fig. 2B are the result of stimulating the cells with 1 *μ*M of staurosporine for 8 h. The quadrants for Fig. 2B show a very similar pattern to the quadrants of Fig. 2A indicating that both MK886 and staurosporine induced apoptosis result in an exposure of phosphatidylserine. In addition to discriminating the population of cells from each other, the double staining enables flow cytometry gating as a function of fluorescent intensity and thus a separation for further analysis of the apoptotic and non-apoptotic cell populations.

### MK886 and staurosporine-induced apoptosis is mitochondrial mediated

**Figure 3.**
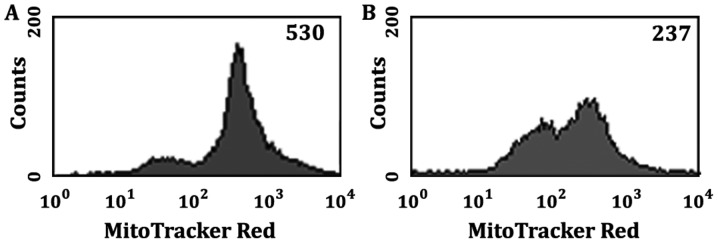
Changes in mitochondrial membrane function due to apoptosis: histograms represent fluorescence intensity of Mito Tracker Deep Red 633 dye where a decrease in fluorescence indicates a decrease in mitochondrial membrane function which is indicative of a mitochondrial mediated apoptotic pathway. The histogram of (A) with the higher median fluorescent intensity (MFI) of 530 represents MFI of DMSO vehicle control cells. The lower valued MFI histogram shown in (B) 237 represents a monolayer of LN18 cells treated with MK886 for 8 h.

Changes in mitochondrial function are early events in the pharmacological induction of apoptosis. The histograms of Fig. 3 represent fluorescence intensity of Mito Tracker Deep Red 633 dye versus cell count. The Mito Tracker histograms show cells that were DMSO vehicle controls (Fig. 3A) and cells that were treated with 50 *μ*M MK886 for 8 h (Fig. 3B). Cells were harvested and labeled with Mito Tracker Deep Red 633 dye and analyzed by flow cytometry as outlined in Materials and methods. It can be seen that the intensity of the emissions of the mitochondria bound Mito Tracker Deep Red decreases as the monolayer of LN18 cells proceed through apoptosis. For Fig. 3 the median fluorescent intensity (MFI) of the DMSO vehicle control LN18 cells is 530 (Fig. 3A) whereas the MK886 treated cells show a decrease in average intensity to a value of 237 (Fig. 3B). Mito Tracker Deep Red is a membrane potential-dependent fluorescent dye that becomes permanently bound to the mitochondria, and remains attached after the cell dies or is fixed. Downstream events of mitochondrial pore activation leading to programmed cell death are associated with changes in mitochondrial membrane potential. A decrease in fluorescence intensity indicates a decrease in mitochondrial membrane function which is a telltale sign that apoptosis is occurring via a mitochondrial pathway. Similar results were obtained for staurosporine indicating staurosporine induced apoptosis is also mitochondria mediated (not shown).

### LN18 cells manifest nucleosome release with an intact plasma membrane

**Figure 4.**
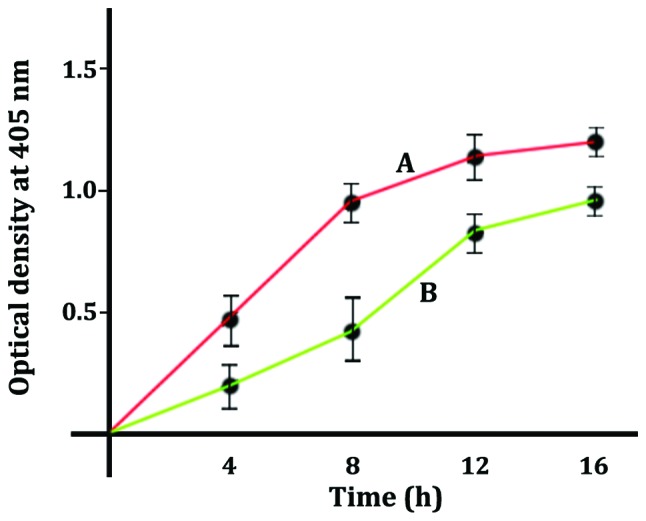
Nucleosome release upon treatment of LN18 cells with staurosporine or MK886. Plots show the absorbance at 405 nm vs. time for a monolayer of LN18 cells stimulated with 1 *μ*M of staurosporine (curve A, red) or 50 *μ*M of MK886 (curve B, green). Increase in absorbance is indicative of increased histone/nucleosome release into the cytoplasm.

Apoptosis as evidenced by nuclear disintegration and the presence of soluble DNA/histone complexes as nucleosomes released from the nucleus is illustrated in Fig. 4. LN18 cells were treated with 1 *μ*M of staurosporine (curve A, red) or 50 *μ*M of MK886 (curve B, green). The plots of Fig. 4 were generated by a quantitative sandwich-enzyme-linked-immunoassay as described in the Materials and methods. The increases in optical density indicate an increase in the presence of mono- and oligonucleosomes in the cytoplasmic fraction of the permeabilized cells. The optical densities of Fig. 4 are presented as the values minus the blank. Blank values for the cells that were permeabilized, but not treated with inducing agent, gave an optical density of <0.1. The supernatants from the samples that were not permeabilized gave optical densities that were barely detectable indicating the detected nucleosomes in curves A and B were released from the nucleus by an apoptotic process. The time course shows that staurosporine treatment (curve A, red) releases the DNA histone complexes at a faster rate than the MK886 treated cells (curve B, green). When comparing staurosporine to MK886, data points of the two curves A and B were shown to be different by the t-test with a p-value <0.05 accepted as a significant difference.

### Cleaving double stranded DNA during late apoptosis

**Figure 5.**
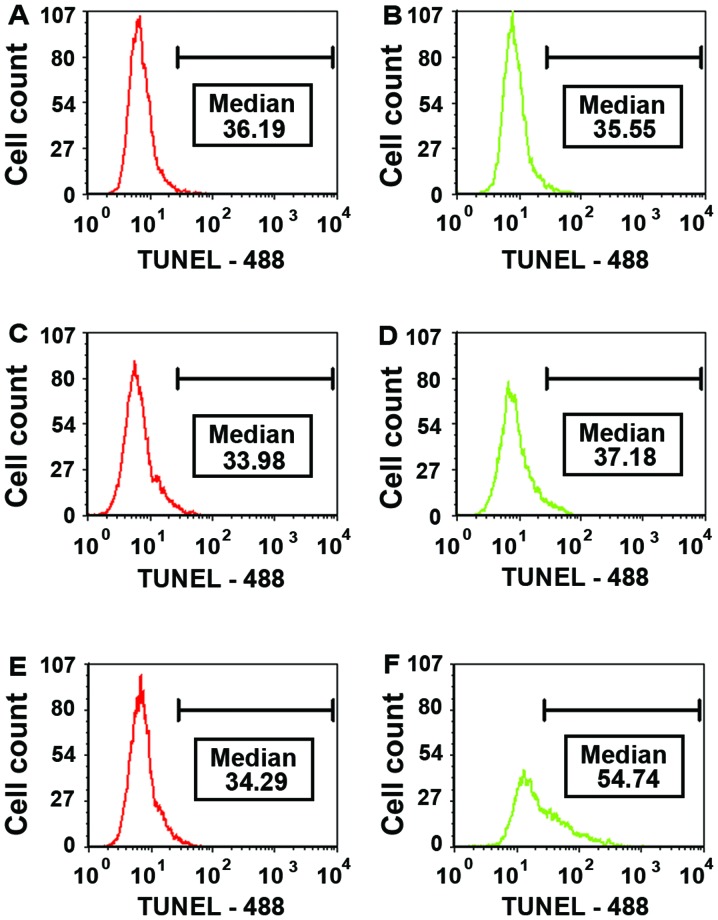
Time course of TUNEL assay for apoptotic LN18 cells. Histograms illustrate the increase in terminal deoxynucleotidyl transferase dUTP nick end-labeling (TUNEL) over time for monolayers of LN18 cells treated with 1 *μ*M of staurosporine (B, D and F, green) compared to non-treated DMSO vehicle control cells (A, C and E, red). The time points are 0 h (A and B), 6 h (C and D), and 15 h (E and F). The median fluorescent intensity (MFI) readings shown in the panels are the median 488 fluorescent outputs of cell populations gated for outputs above 20 intensity units. At 15 h there is a shift up in the gated 488 MFI of 54.74 (F) indicating an increase in the cell population with more DNA fragmentation which is characteristic of late apoptosis.

**Figure 6.**
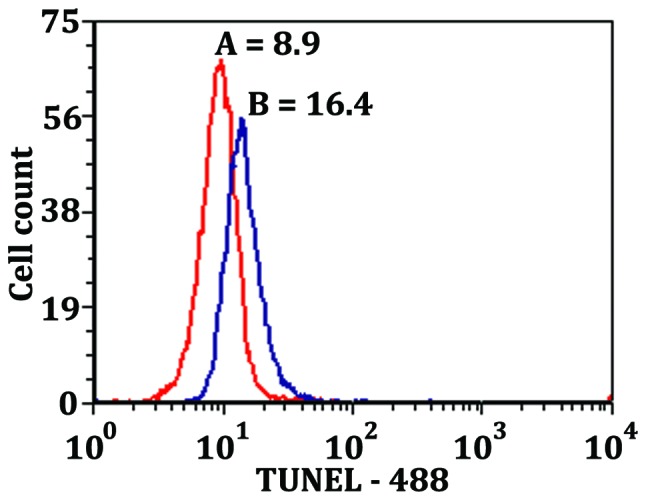
TUNEL assay for gated apoptotic and non-apoptotic cell populations: the histograms show the TUNEL-488 intensity versus cell count for a monolayer of LN18 cells treated with 50 *μ*M of MK886 for 12 h. The population of cells were gated for non-apoptotic cells (plot A, red) and apoptotic cells (plot B, blue). The value of plot A is the total median fluorescent intensity (MFI) of 8.9 for the non-apoptotic cell population. Plot B indicates the MFI for the apoptotic cell population showing a value of 16.4.

Terminal Uridine Nick End-Labeling (TUNEL) is an assay for detecting DNA fragmentation due to apoptosis. The TUNEL assay is designed to detect late apoptosis. Extensive fragmentation of nuclear DNA that generates a large number of DNA double-strand breaks is one of the most characteristic events of late apoptosis. The 3′OH-termini of the 2′-deoxyuridine 5′-triphosphate (dUTP) nicks serve as primers and become labeled in this procedure with BrdU when incubated with Br-dUTP in a reaction catalyzed by the exogenous terminal deoxynucleotidyl transferase (TdT). The histograms in Fig. 5 show the MFI for the cell population gated for readings above 20 intensity units. Fig. 5A, C and E demonstrate the TUNEL histograms for DMSO vehicle control LN18 cells over a time period of 15 h while Fig. 5B, D and F illustrate the histograms resulting from treating the LN18 cells with 1 *μ*M of staurosporine over the same time period. There was no significant change in the DMSO vehicle control cell population over the 15 h. Conversely, Fig. 5D shows a shift of the cell population with corresponding higher median fluorescent intensity (MFI) (54.74) indicating an increase in DNA double-strand breaks as the cells proceed through apoptosis. The histograms of Fig. 6 show the TUNEL-488 intensity vs. cell count for LN18 cells treated with 50 *μ*M of MK886 for 12 h. Plot A of Fig. 6 is the TUNEL results of the non-apoptotic cell population showing MFI of 8.9. Plot B of Fig. 6 shows the TUNEL results of apoptotic population showing MFI twice as high as the non-apoptotic population (16.4 vs. 8.9). These results demonstrate that there is more DNA fragmentation in the apoptotic population of the MK886 treated cells.

### Reduction of HLA-ABC, EGFR, IGF1R, IGA3 and IGB4 cell surface cluster determinants during apoptosis

**Figure 7.**
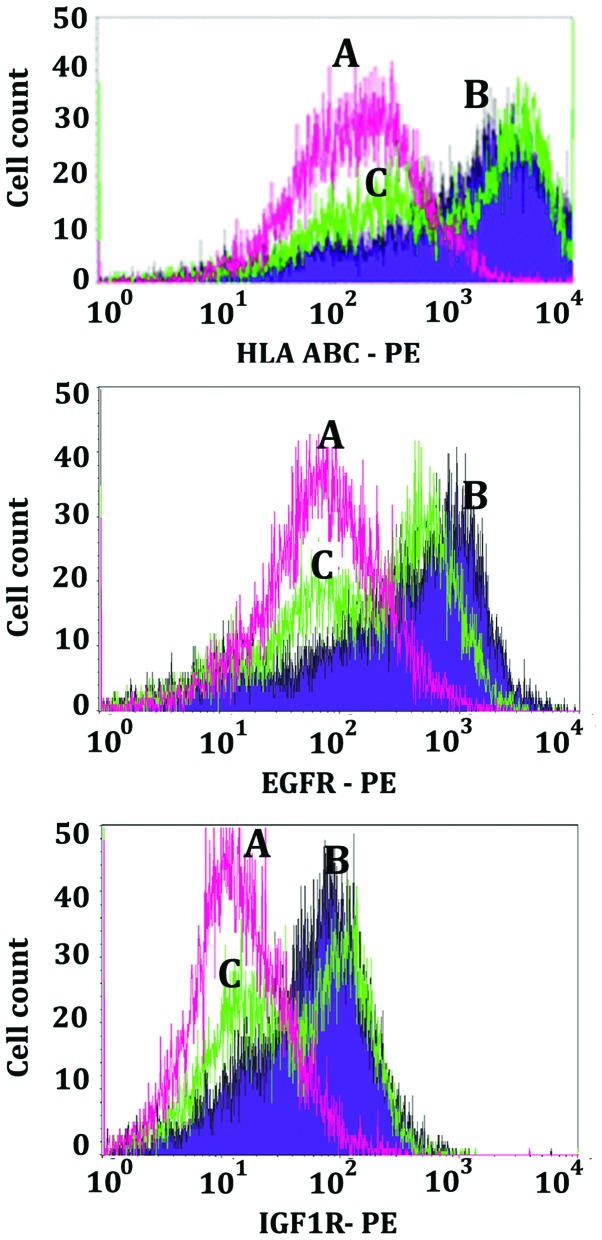
Apoptotic decreases in HLA, EGFR and IGF1R cell surface determinants. Composites (curves A, pink; curves B, purple; and curves C, green) of flow cytometry histograms showing phycoerythrin fluorescence intensity vs. cell count for the LN18 histocompatibility antigens HLA-ABC, and cell surface growth factor receptors EGFR and IGF1R. All curves were obtained by using mouse primary antibodies followed by goat anti-mouse-IgG conjugated to phycoerythrin. Curves labeled A (pink) show the non-specific labeling that was obtained by using mouse anti-KLH as a primary antibody. Curves labeled B (purple) indicate the fluorescence obtained by the mouse antibodies specific for the determinants noted in the abscissae for the non-treated vehicle control (DMSO) LN18 cells. Curves labeled C (green) indicate the fluorescence obtained by the mouse antibodies specific for each of the determinants noted in the abscissae for those LN18 cells treated with MK886 for 14 h.

**Figure 8.**
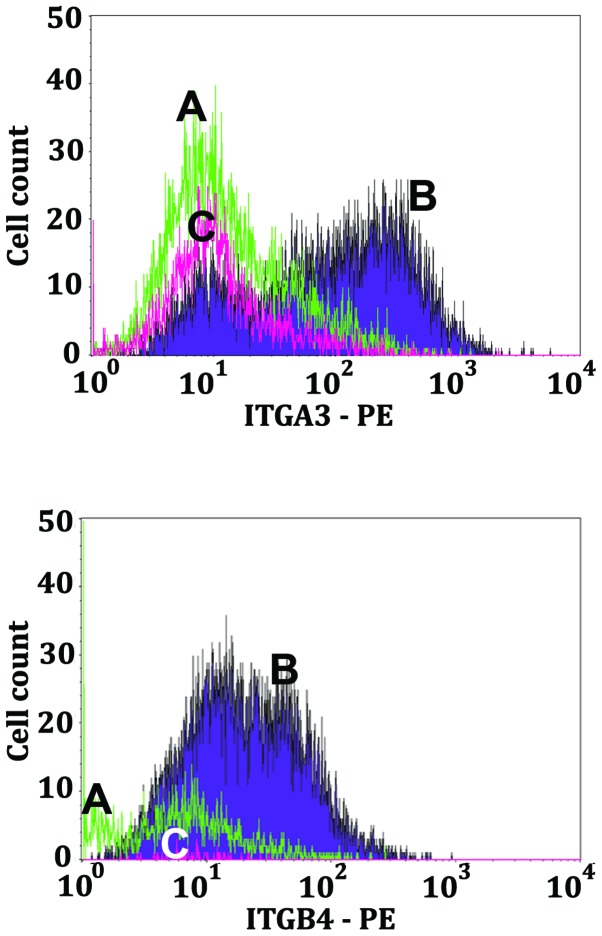
Apoptotic decreases in INTα3 (IGA3) and INTβ4 (IGB4) cell surface determinants. Composites (curves A, green; curves B, purple; and curves C, pink) of flow cytometry histograms showing phycoerythrin fluorescent intensity vs. cell count for the LN18 cell surface integrins α3 (IGA3) and β4 (IGB4). All curves were obtained by using mouse primary antibodies followed by goat anti-mouse-IgG conjugated to phycoerythrin. Curves labeled A (green) show the non-specific labeling that was obtained by using mouse anti-KLH as a primary antibody. Curves labeled B (purple) indicate the fluorescence obtained by the mouse antibodies specific for each integrin for the non-treated vehicle control (DMSO) LN18 cells. Curves labeled C (pink) indicate the fluorescence obtained by the mouse antibodies specific for each integrin when the LN18 cells were treated with MK886 for 14 h.

**Figure 9.**
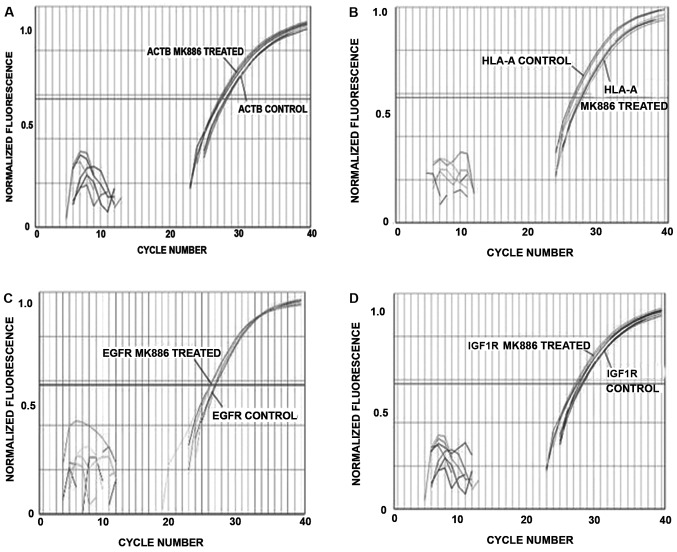
Measurement of steady state mRNA expression for ACTB, HLA-A, EGFR and IGF1R. Data were accumulated by real-time RT-PCR. cDNA(s) were generated from RNA isolated from both MK886 (10 h) and vehicle control (DMSO) LN18 calls. PCR amplicons were generated with primer probes that were specific for ACTB, HLA-A, EGFR and IGF1R. The abscissae show the progression of cycles up to a maximum of 40. The intersections of the horizontal threshold lines indicate the number of cycles to threshold (Ct). The ordinate values indicate the fluorescence normalized to the value at 40 cycles.

Figs. 7 and 8 are examples of the decrease in density of cell surface determinants during apoptosis as measured by flow cytometry. Monolayers of LN18 cells were either not treated control cells (purple, curves B) or treated for 14 h with 50 *μ*M MK886 (green, curves C). Following treatment, cells were harvested and prepared to be analyzed by flow cytometry as described in the Materials and methods. Non-reacting mouse monoclonal anti-KLH was used as the primary antibody negative control for curves A (pink). The histograms of Fig. 7 illustrate that as the LN18 cells proceed through apoptosis there is a decrease in the cell surface determinants of Class-1 histocompatibility markers HLA-ABC (top panel of Fig. 7), epidermal growth factor receptor (EGFR, middle panel of Fig. 7), and insulin growth factor 1 receptor (IGF1R, lower panel of Fig. 7). The bimodality of the apoptotic cell populations (green, curves C) indicate that 14 h after the stimulation of the LN18 monolayer with MK886 the cells exist as a mixture of apoptotic and non-apoptotic cells. This was verified by gating the apoptotic cell population for those that were both Annexin V-488 and 7-AAD positive as compared to those that were Annexin V-488 positive and 7-AAD negative thus comparing early and late apoptosis. For those cells gated for early apoptosis the histogram had the appearance of the higher intensity (right portion only) of curve C (data not shown). On the other hand, the cells that were gated for late apoptosis produced the single histogram of the lower intensity left portion of curve C (data not shown). The fact that the apoptotic stimulated cells produce a cell population of varying degrees of apoptosis is relevant to the view that stimulated LN18 cells can exist as a mixture of apoptotic and viable non-apoptotic cells. This was also borne out by the dot plots of Fig. 2. In a similar manner to Fig. 8, the histograms of Fig. 9 illustrate that as the LN18 cells proceed through apoptosis there is a decrease in the LN18 cell surface integrins α3 (IGA3, top panel of Fig. 9) and β4 (IGB4, middle panel of Fig. 9).

### Transcriptions of mRNA for integrins, HLA, EGFR, IGF1R, IGA3 and IGB4 are not decreased during apoptosis as measured by RT-PCR

**Figure 10.**
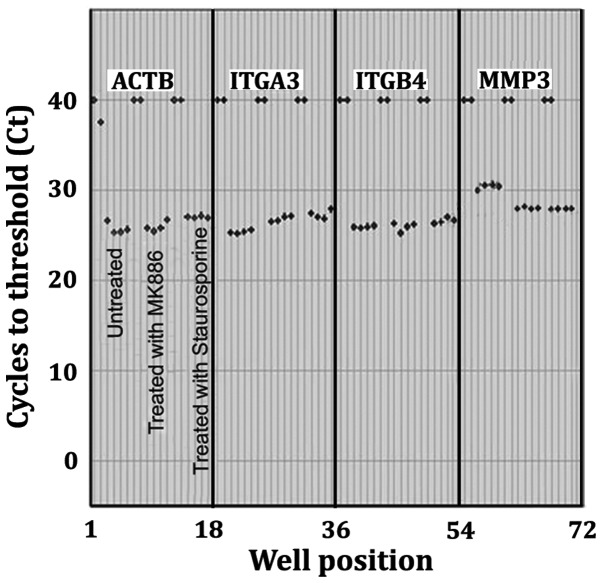
Measurement of steady state mRNA expression for INTα3 (IGA3), INTβ4 (IGB4) and MMP-3. Data show the steady state mRNA expression for the β-actin (ACTB) control for the integrins α3 (IGA3), β4 (IGB4) and the metalloproteinase MMP-3. Cycles to threshold (Ct) were obtained from the fluorescence vs. cycle number curves intersecting with a fixed threshold line where the threshold line was set to intersect at the log phase of the curves (not shown). There are 2 replicate blanks and 4 replicate samples for each gene tested under the conditions of untreated control (DMSO) LN18 cells, and cells treated with MK886 for 10 h. The duplicate blank controls devoid of reverse transcriptase for those samples that did not reach threshold within 40 cycles are presented as having Ct values of 40. The displayed Ct values for the 4 replicates of each sample indicate the level of mRNA for the untreated and MK886 treated LN18 cells.

Real-time RT-PCR was used to determine if steady state mRNAs for integrins, HLA, EGFR and IGF1R are expressed in LN18 cells. Fig. 9 is a plot of the normalized fluorescence vs. the sample well position for cycling DMSO vehicle control cells and 50 *μ*M MK886 treated LN18 cells. Fig. 9A shows that there is significant expression for the housekeeping gene β-actin (ACTB), HLA (Fig. 9B), EGFR (Fig. 9C) and IGF1R (Fig. 9D). The panels of Fig. 9 show that there is no decrease in the steady state mRNA at 10 h, which is the beginning of the time period when the proteins of HLA, EGFR and IGF1R are decreasing. The RT-PCR results thus indicate that the downturn in these proteins is due to enzymatic degradations and not reduced transcription. Measurement of mRNA expression by RT-PCR for β-actin (ACTB), α3 (IGA3), β4 (IGB4) and MMP-3 are shown in Fig. 10. PCR amplicons were generated from RNA isolated from DMSO vehicle control LN18 cells, MK886 treated or staurosporine treated LN18 cells. Cycles to threshold (Ct) were obtained from the fluorescence vs. cycle number curves intersecting with a fixed threshold line where the threshold line was set to intersect at the log phase of the curves (not shown). There are 2 replicate blanks and 4 replicates for each sample. The total number of cycles to completion of the PCR portion of the experiment was 40. The duplicate blank controls, for which reverse transcriptase was omitted, are presented as having Ct values of 40 because the samples did not reach threshold within 40 cycles. The displayed Ct values for the 4 replicates of each sample indicate the level of mRNA in the DMSO vehicle control LN18 cells as compared to 50 *μ*M MK886 or the 1 *μ*M staurosporine stimulated LN18 cells for 10 h. Sample Ct values being significantly <40 indicate there was a steady state mRNA expression. There were no significant differences in the steady state mRNA expression for the MK886 or staurosporine stimulated cells as compared to the DMSO vehicle control cells for the housekeeping gene ATCB and the integrins IGA3 and IGB4. Furthermore, the ΔCt for all of the samples were not significantly different than the ΔCt for the ACTB control. This indicates that the apoptotic reduction in the integrin determinants is due to enzymatic degradation and not a reduction in transcription. Conversely, there was a significant increase in the steady state levels of mRNA at 10 h stimulation with MK886 or staurosporine as compared to the unstimulated control for the metalloproteinase MMP-3.

### Density of surface MMP-3 for apoptotic and non-apoptotic LN18 cells

**Figure 11.**
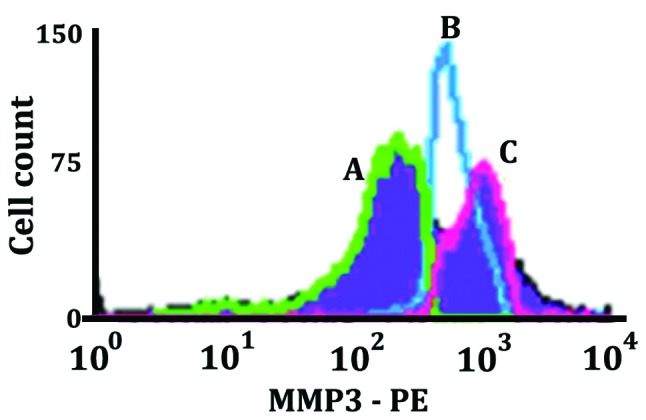
Levels of MMP-3 protein in MK886 treated LN18 cells. The monolayer of LN18 cells was treated with 50 *μ*M MK886 for 8 h. The primary antibody was rabbit IgG directed against the hinge region of MMP-3. Histograms illustrate the cell surface level of MMP-3 as measured by the secondary goat anti-rabbit IgG phycoerythrin fluorescent output. The histograms are a result of gating upon the emissions of normal LN18 cells that were negative for Annexin V and 7-AAD (B, blue), early apoptotic cells that were positive for Annexin V, but negative for 7-AAD (C, pink), and late apoptotic cells that were positive for both Annexin V and 7-AAD (A, green).

MMP-3 is one of the secreted metalloproteinases that can act as a sheddase in addition to effecting paracrine degradation of surface determinants of neighboring cells and can also have a role in degradation of the extracellular matrix. The histogram in Fig. 11 shows the level of MMP-3 expression on different populations of LN18 cells resulting from being stimulated with 50 *μ*M MK886 for 8 h. The histogram labeled B (blue) is the Annexin V(−), 7AAD(−) normal live population of LN18 cells. The histogram labeled C (pink) illustrates a population of cells in early apoptosis [Annexin V(+), 7AAD(−)]. The histogram labeled A (green) shows the population of cells that were in late apoptosis [Annexin V(+), 7AAD(+)]. It can be seen that the density of MMP-3 increases in the apoptotic cell population as compared to the non-apoptotic population, but subsequently decreases in late apoptosis. The data are consistent with what would be expected for a secreted metalloproteinase where there is an initial increase on the cell surface followed by a decrease on the cell surface as the secretion of the MMP-3 progresses.

### Effect of protease inhibitors upon changes in the density of cell surface HLA-A determinants induced by MK886 as measured by flow cytometry

**Figure 12.**
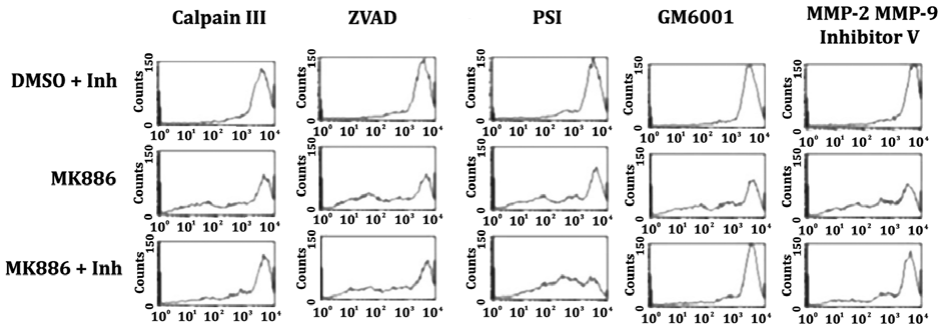
Apoptotic decrease in cell surface determinants prevented by metalloproteinase inhibitors. Flow cytometry histograms showing phycoerythrin fluorescence intensity (abscissa) vs. cell count (ordinate) for the LN18 cell surface HLA-A determinants. All curves were obtained by using mouse primary antibodies against HLA-A determinants followed by F(ab)_2_ goat anti-mouse-IgG conjugated to phycoerythrin. Histograms provide examples of the effects of proteolytic inhibitors upon the reduction of HLA-A cells surface determinants by MK886 induced apoptosis. The headings of the columns show the proteolytic inhibitors used. The labelings of the rows show the treatment of the LN18 monolayer. The histograms of the first row are the control group and illustrate the effect of the proteolytic inhibitors upon surface HLA-A determinants in the untreated DMSO vehicle control cells incubated for 16 h. The histograms of the second row illustrate the effect of upon surface HLA-A determinants in LN18 cells treated with MK886 for 16 h in the absence of proteolytic inhibitors. The histograms of the third row demonstrate the ability of the proteolytic inhibitors to prevent the decreases in HLA-A determinants that can be induced by MK886. For the data of the third row, the protease inhibitors were added 7 h after the MK886 and the reaction was allowed to proceed for an additional 9 h for a total of 16 h. The data of the second row provide 3 separate samples showing a significant decrease in HLA-A surface determinants induced by 16 h treatment with MK886.

The histograms of Fig. 12 provide examples of the effects of proteolytic inhibitors upon the reduction of HLA-A cell surface determinants by MK886 induced apoptosis. The following inhibitors were used: Calpain III (50 *μ*M) inhibits calpain 1 and 2; ZVAD-FMK (50 *μ*M) a broad caspases inhibitor; PSI (50 *μ*M) proteosome inhibitor that also inhibits NF-κB and chymotrypsin; GM6001 (50 *μ*M) a broad matrix metalloproteinase inhibitor; and, MMP-2 MMP-9 Inhibitor V (50 *μ*M) a broad matrix metalloproteinase inhibitor with an IC_50_ that is lower for MMP-2 and MMP-9. The histograms of the first row are the control group and illustrate the effect of the proteolytic inhibitors upon surface HLA-A determinants in the DMSO vehicle control cells that were incubated for 16 h. The histograms of the second row illustrate the effect upon surface HLA-A determinants in LN18 cells treated with 50 *μ*M MK886 for 16 h in the absence of proteolytic inhibitors. The histograms of the third row demonstrate the ability of the proteolytic inhibitors to prevent the decreases in HLA-A determinants that can be induced by MK886. For the histograms of the third row, the protease inhibitors were added 7 h after the MK886 and the reaction was allowed to proceed for an additional 9 h for a total of 16 h. The histograms of the first row demonstrate that the inhibitors in the absence of the MK886 have similar levels of HLA-A expression. The 5 histograms of the second row show a significant decrease in HLA-A surface determinants induced by 16 h treatment with MK886. The histograms of the third row show that the metalloproteinase inhibitor GM6001 (column 4) and the metalloproteinase inhibitor MMP-2, MMP-9 Inhibitor V (column 5) prevent the HLA-A reduction induced by MK886 providing evidence that active metalloproteinases are required for the apoptotic degradation of the cell surface determinants. The protease inhibitor calpain III had little effect upon the apoptotic degradation of the HLA-A determinants (column 1 of row 3). The broad caspases inhibitor ZVAD-FMK added 7 h after the MK886 had no discernible effect (column 2 of row 3) indicating that the caspase cascade of the apoptotic process has taken place prior to the apoptotic degradation of the cells’ surface determinants. The proteosome inhibitor PSI (column 3 of row 3) exacerbated the HLA-A degradation possibly due to an enhancement of the apoptotic process induced by MK886.

## Discussion

There have been many important foundational studies defining the intrinsic intracellular mechanisms of apoptosis. Additionally, there has been a great deal of interest in the apoptotic effects of modulating receptor mediated transduction mechanisms. Comparatively, there has been little emphasis on determining the functionally opposite process of modulating the cell’s surface receptors as the cells progress through apoptosis. An objective of this investigation was to use the LN18 cell line as a model to examine the apoptotic modulation of cell surface integrins, EGFR, IGF1R and MHC-1. Our first interest was to correlate the state of apoptosis with the degradation of these cell surface determinants. In the context of the study’s objectives, it was essential to establish the time course and characterize aspects of MK886 and staurosporine induced apoptosis in the LN18 cells. Although the pharmacological agents MK886 and staurosporine have different modes of apoptotic initiation, both agents stimulate apoptosis intrinsically and soon after initial stimulation continue on a pathway of apoptosis that is mitochondrial mediated. Intrinsic apoptosis does not require transmembrane receptor activation, but rather is the result of intracellular signals that act directly on targets within the cell and is typically mitochondrial mediated. Pathways of mitochondrial mediated apoptosis have been extensively studied and are becoming ever more well-known. Pro-apoptotic proteins of the Bcl-2 family (40,41) promote the increase in mitochondrial membrane permeability (42) with a loss of mitochondrial membrane potential (43) whereupon the mitochondrial proteins cytochrome c (44) and Smac/DIABLO (45) are released into the cytosol which activate the apoptotic promoting construct Apaf-1 (apoptotic protease activating factor 1) (46) as well as procaspase-9 (47) and concurrently disrupts apoptotic inhibition normally brought about by IAPs (inhibitors of apoptosis) (48). An integral part of the apoptotic cascade is the activation of effector caspases (49,50) which control downstream processes that eventually result in the release of nucleosomes into the cytosol and the cleavage of double stranded DNA (51–53). The dot plots, Mito Tracker, nucleosome release and TUNEL experimentation of this present report are in agreement with the established pathways of caspases mediated intrinsic apoptosis. Despite this, the broad caspases inhibitor ZVAD-FMK, when added 7 h after the induction of apoptosis, had no effect on the degradation of integrins, EGFR, IGF1R and MHC-1 which takes place 7–15 h after the induction of apoptosis. This is an indication that caspases are not the terminal proteases that degrade the CDs and further implies that the caspase cascade involving relevant proteases is actuated before the CDs are proteolytically degraded.

Accompanying the enzymatic cascade are morphological changes that are typical of pharmacologically induced apoptosis which included rounding and shrinking of the cells resulting in a separation of the cells from each other and from the extra-cellular matrix. It is well established that the loss of integrin signaling by detachment of cells from the extracellular matrix reduces survival signals and can promote an anoikic form of apoptosis (17,54). As aforementioned, the question explored here is, when apoptosis is induced by mitochondrial mediated intrinsic pathways does the apoptotic process itself inherently degrade cell surface receptors that are likely to disrupt transmembrane integrin and growth factor receptor signaling? Certainly the separation of cells from each other and separation of the cells from the extracellular matrix, which occurs during apoptosis, is indicative of disrupting the heterodimeric transmembrane integrin receptor signaling that normally occurs. More specifically, the data presented here show that α-β components of integrins are downgraded during apoptosis. Integrin mediated signaling pathways initiated by the ligation of matrix proteins induce clustering and the phosphorylation of pp125FAK (focal adhesion kinase) which leads to its association with other kinases and adapter molecules, particularly PI 3-kinase (Akt), which in turn leads to activation of downstream survival pathways (55,56). IGF1R and EGFR are among growth factor receptors in which transduction mechanisms involve transmembrane receptor tyrosine kinases which affect the recruitment and activation of intracellular effector proteins that promote cell survival (13,57,58). As with the integrins, the flow cytometry data presented here demonstrate that as the LN18 cells progress through apoptosis there are reductions in the antigenic determinants of the growth factor receptors IGF1R and EGFR. Thus, the simultaneous degradation of integrins, IGF1R and EGFR would concurrently disrupt the critical role that they have in the kinase cascades of adhesion and growth factor regulated survival signaling. The apoptotic disruption of growth factors and integrins simultaneously is relevant to circumventing apoptotic resistance particularly in the context of the interrelationships and cross-talk that exist between growth factor/growth factor and integrin/growth factor intracellular signaling systems (14,15,59).

It was determined by Real-time RT-PCR that the steady-state transcription of integrins and growth factor receptors did not decrease significantly during the time span when the reduction in the integrins, EGFR, IGF1R and MHC-1 occurred. GM6001 and MMP Inhibitor V effectively prevented the apoptotic down regulation of class 1 histocompatibility antigens (HLA-A) even when added 7 h after the induction of apoptosis. Consequently, it is likely that integrins, EGFR, IGF1R and MHC-1 are not being reduced by a decrease in transcription but rather by proteases with the implication that metalloproteinases are part of the effector proteases. It has been known for some time that metalloproteinases play an important role in the degradation of the extracellular matrix and cell surface proteins (60,61). In humans, there are 23 known members of the matrixin metalloproteinases most of which are capable of acting as sheddases and include: matrix metalloproteinases (MMPs) (62,63), membrane type matrix metalloproteinases (MT-MMPs) (64,65), a disintegrin and metalloproteinases (ADAMs) (66) and a disintegrin and metalloproteinases with thrombospondin type 1 motif (ADAMTs) (67). This study has not determined which of the metalloproteinases are effector proteases, nor was it determined how the apoptotic process converts the zymogen forms of metalloproteinases to their active form. Nevertheless, there is ample evidence in the literature that latent precursor forms of metalloproteinases can be processed into biologically active forms by furin and PC5 and possibly other members of the family of proprotein convertases (68–70). Furin convertase can proteolytically process a variety of precursor forms of molecules including transforming growth factor β1 (TGFβ1) (71,72). TGFβ1 is immunosuppressive (73,74) and can be protective in various pathologies including autoimmune diseases (75). However, one of the unsettling conditions of glioblastoma patients is the high frequency of immunosuppression (76,77) with mounting evidence that the immunosuppression involves furin processing of TGFβ (78,79). As far as we know, none of the studies have examined the interrelationship of furin and the activation of metalloproteinases and TGFβ in glioblastoma cells during the process of apoptosis. In light of these implications and the data presented here showing enhanced metalloproteinase activity during apoptosis, a study of furin activation of metalloproteinases and TGFβ in apoptotic glioblastoma cells may be a pursuit worthy of undertaking.

In this study, MHC-1 antigenic levels are reduced when the cells proceed through the apoptotic process. At first thought it may be puzzling to deduce any positive effect of having a reduction of MHC determinants in the commitment to apoptosis or to the elimination of apoptotic cells. Natural killer cells (NK cells) pass the blood-brain barrier and infiltrate the brain whereupon they are modified by resident immune cells and target virally infected cells and tumor cells rendering the role of infiltrating NK cells critical as part of the brain’s innate immune system (80). Actually, NK cells demonstrate enhanced cytotoxic activity towards those cells missing or possessing low MHC-1 self-markers (81,82) which should make them effective eliminators of those glioblastoma cells that have low levels of MHC markers. Consequently, the data showing activation of metalloproteinases and the degradation of cell surface proteins during apoptosis of glioblastoma cells has the implications of affecting innate immunity, the extra cellular matrix and the metalloproteinase dependent invasiveness of glioblastoma tumors.

There is renewed interest in treating glioblastomas by enhancing innate and adaptive immunity (83) while including the chemotherapeutic targeting of growth factor receptor (84) and integrin function (85). The optimal standard in the clinical treatment of brain tumors is to kill the tumor cells effectively and completely, without inflammation, while keeping normal brain cells intact. The data presented here are compatible with the opinion that apoptosis normally proceeds in a manner that inhibits integrin and growth factor survival signals while stimulating the brain’s natural immune system. Unfortunately, malignant cells possess mechanisms to escape apoptosis while sustaining growth factor survival signals even in the absence of receptor stimulation along with suppressing the active immune system. Notwithstanding this, the data presented here have enabled us to formulate a perspective for further study that includes the idea that the final stages of the pharmacological induction of apoptosis, to proceed to a full commitment to non-necrotic cell death, involves the degradation of integrin, insulin and epidermal growth factor receptors caused by a programmed dysregulation of the cell’s metalloproteinases.
